# BRCA1 in the DNA damage response and at telomeres

**DOI:** 10.3389/fgene.2013.00085

**Published:** 2013-06-21

**Authors:** Eliot M. Rosen

**Affiliations:** ^1^Department of Oncology, Georgetown University School of MedicineWashington, DC, USA; ^2^Department of Biochemistry, Molecular and Cellular Biology, Georgetown University School of MedicineWashington, DC, USA; ^3^Department of Radiation Medicine, Georgetown University School of MedicineWashington, DC, USA

**Keywords:** breast cancer susceptibility gene 1, DNA damage response, telomeres, ataxia-telangiectasia mutated, homology-directed repair, base excision repair, DNA damage signaling

## Abstract

Mutations of the breast and ovarian cancer susceptibility gene 1 (BRCA1) account for about 40–45% of hereditary breast cancer cases. Moreover, a significant fraction of sporadic (non-hereditary) breast and ovarian cancers exhibit reduced or absent expression of the BRCA1 protein, suggesting an additional role for *BRCA1* in sporadic cancers. *BRCA1* follows the classic pattern of a highly penetrant Knudsen-type tumor suppressor gene in which one allele is inactivated through a germ-line mutation and the other is mutated or deleted within the tumor. BRCA1 is a multi-functional protein but it is not fully understood which function(s) is (are) most important for tumor suppression, nor is it clear why *BRCA1-mutations* confer a high risk for breast and ovarian cancers and not a broad spectrum of tumor types. Here, we will review BRCA1 functions in the DNA damage response (DDR), which are likely to contribute to tumor suppression. In the process, we will highlight some of the controversies and unresolved issues in the field. We will also describe a recently identified and under-investigated role for BRCA1 in the regulation of telomeres and the implications of this role in the DDR and cancer suppression.

## Introduction

The breast and ovarian cancer susceptibility gene 1 *(BRCA1)* on chromosome 17q21 was identified and cloned in 1994 by Miki et al. (1994), 1 year before the reported cloning of a second breast cancer susceptibility gene *(BRCA2)* on chromosome 13q12-13 by Wooster et al. (1995). The *BRCA1* gene fits the classical Knudsen “two hit” model of a tumor suppressor gene. This model was developed by Dr. Alfred Knudsen, Jr. in 1971 and was first applied to understand the genetics of retinoblastoma, a tumor of the cells of the retina in the eye that occurs in very young children. According to this model, a cell requires two “hits” (mutations), one in each allele of a tumor suppressor gene (e.g., RB1, the retinoblastoma susceptibility gene) for a cancer to develop. In hereditary cancers, the first “hit” is a germ-line mutation, which is thus present in all somatic cells. The second “hit” (often the deletion of a portion of the chromosome containing the wild-type allele) occurs only in somatic cells within the target tissue, and leads to cancer. In this model, the inheritance pattern is autosomal dominant (since only one mutant allele is inherited). However, at the molecular level, the tumor exhibits a “recessive” pattern, since both alleles must be inactivated for a tumor to occur. In the case of *BRCA1*, women inherit one mutant allele and one wild-type allele; but in nearly all tumors that develop in BRCA1-mutation carriers, the wild-type allele is lost (Merajver et al., 1995), leaving no functional BRCA1 in the tumor cells.

Although inherited BRCA1-mutations account for a very small proportion of all breast cancers (2.5–5%), a significant proportion of the much larger group of sporadic (non-hereditary) breast cancers (30–40%) exhibit absent or significantly reduced levels of BRCA1 protein, suggesting that loss of BRCA1 function whether by epigenetic silencing, mutation, or other mechanisms is a common component in the pathogenesis of sporadic breast cancer (Rice et al., 1998; Taylor et al., 1998; Wilson et al., 1999; Esteller et al., 2000; Staff et al., 2003). Consistent with the Knudsen model, inactivating mutations of both *BRCA1* alleles are uncommon in sporadic breast cancers, since the probability of two acquired hits in a somatic cell is much lower than that of a second hit in a cell that has already acquired the first hit by inheritance.

## The human *BRCA1* gene and protein

**Figure 1:**
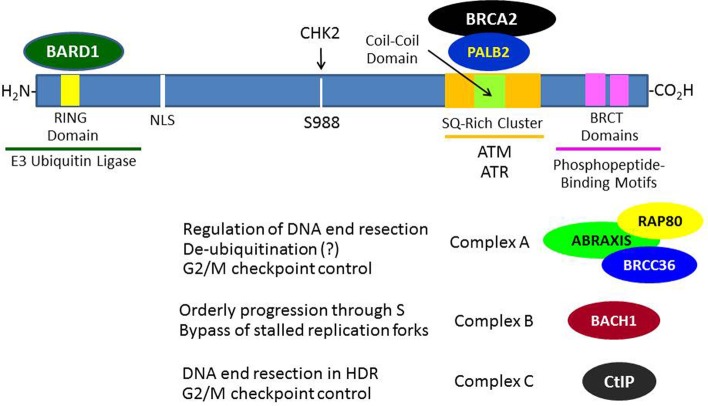
**Breast and ovarian cancer susceptibility gene 1 protein interactions that contribute to its role in the DNA damage response**. In response to DNA damage, BRCA1 is phosphorylated at various sites by several kinases (e.g., ATM, CHK2, and/or ATR) and forms several different types of complexes that are recruited to the sites of DNA damage through various mechanisms. The roles of these complexes in DNA damage signaling and repair are only partially understood. The formation of these BRCA1 complexes is dependent upon the mutually exclusive interactions of its BRCT domains with phosphorylated motifs within Abraxis, BACH1, or CtIP. BRCA1 functions to recruit BRCA2 to DNA damage sites through an intermediary protein, PALB2 (partner and localizer of BRCA2). The interaction of the BRCA1 N-terminal RING domain with its binding partner BARD1 is required for tumor suppression, since BRCA1-mutations that disrupt this interaction lead to cancer.

The BRCA1 gene contains 24 exons, 22 of which are coding exons and 2 of which are non-coding (Miki et al., 1994). Exon 11 is the largest exon and encodes about 60% of the protein. The BRCA1 protein consists of 1863 amino acids, migrates on SDS-PAGE at a molecular mass (M_r_) corresponding to 220 kDa, and does not show significant structural homology to other human proteins with the exception of an N-terminal RING domain (amino acid 20–64) and a C-terminal acidic domain (TAD). This TAD can mediate transcription when ligated to a suitable DNA-binding domain (Monteiro et al., 1996). The C-terminal TAD of BRCA1 contains a tandem repeat of 95 amino acids each called a BRCA1-associated C-terminal (BRCT) domain that is homologous to similar domains found within various DNA repair and cell cycle checkpoint proteins (Bork et al., 1997) (see Figure 1). The BRCT domains were subsequently found to be phosphoprotein-binding modules that bind to specific phosphoserine- or phosphotyrosine-containing motifs and are involved in the processes of DNA damage signaling and repair (Manke et al., 2003). The BRCA1 RING domain was found to interact with another RING-containing protein BRCA1-associated ring domain 1 (BARD1) protein to mediate an enzymatic function as an E3 ubiquitin ligase (to be discussed below in detail). The BRCA1 protein also contains functional nuclear import and nuclear export signals, suggesting that it may shuttle between the nucleus and cytoplasm, although it seems that most BRCA1 functions occur within the nucleus (Rodríguez and Henderson, 2000).

Breast and ovarian cancer susceptibility gene 1 has been found to regulate the activity of a variety of different transcription factors although BRCA1 is not itself a sequence-specific DNA-binding transcription factor. The usual paradigm is that BRCA1 binds directly to many different transcription factors [e.g., p53, estrogen receptor, progesterone receptor, androgen receptor, STAT1, c-Myc, NF-κ B, octamer-binding transcription factor 1 (OCT1), and others], while other portions of the BRCA1 molecule make contact with components of the basal transcription machinery (RNA polymerase II holoenzyme) and/or with components of chromatin remodeling complexes (reviewed in Rosen et al., 2006). In this context, BRCA1 functions as a transcriptional co-regulator that may either stimulate (e.g., p53, androgen receptor, OCT1) or inhibit (e.g., estrogen receptor, progesterone receptor, c-Myc) transcriptional activity. Thus, some BRCA1 functions are linked to the regulation of transcription, although which of these may contribute to tumor suppression remains unclear to date.

## Atm-dependent signaling and the DNA damage response

**Figure 2:**
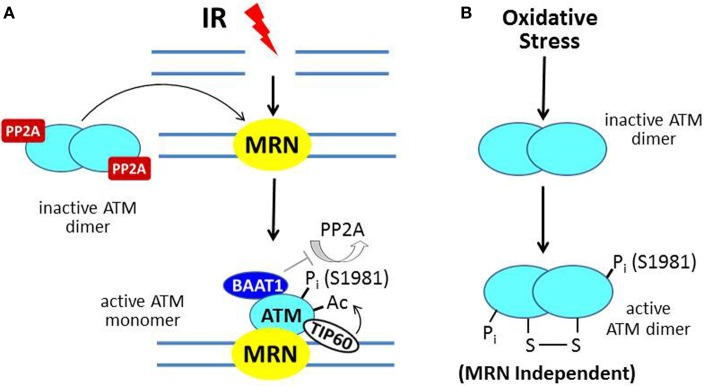
**ATM activation by ionizing radiation (IR) vs. oxidative stress. (A)** In IR-induced activation, the MRN complex, a DNA damage sensor, is recruited to DSBs; and MRN then recruits ATM. In undamaged cells, ATM is a dimer held in the inactive state by PP2A. After IR, PP2A dissociates from ATM, allowing autophosphorylation on S1981 and conversion to a monomer at the MRN/DSB site. The protein BAAT1 binds to activated ATM and prevents dephosphorylation by PP2A. Another step in ATM activation involves binding of TIP60 to chromatin near the DSB and acetylation of ATM, which is required for its full activation. **(B)** In response to oxidative stress, ATM is directly oxidized, forming a disulfide-linked dimer, which is phosphorylated on S1981 and activated.

Ataxia-telangiectasia (A-T) is an autosomal recessive hereditary disorder characterized by neurodegeneration (including cerebellar ataxia), immunodeficiency, predisposition to develop cancer, skin abnormalities, and increased sensitivity to ionizing radiation (IR). A-T is due to mutation of the *ATM* (A-T mutated) gene, the protein product of which functions as a master regulator of the DNA damage response (DDR) (Lavin and Kozlov, 2007). ATM-deficient cells exhibit hypersensitivity to IR and defects in DNA damage-responsive cell cycle checkpoints (see below). The prototypic activator of ATM is the DNA double-strand break (DSB) due to IR. In the model shown in Figure 2A, the broken DNA ends are recognized by the MRN complex of three proteins [MRE11-RAD-50-Nijmegen breakage syndrome 1 (NBS1)], which functions as a DNA damage sensor and translocates to the site of the DSB (Lee and Paull, 2005). ATM normally exists as an inactive dimer which is maintained in that state by the protein serine/threonine phosphatase PP2A (Bakkenist and Kastan, 2003; Goodarzi et al., 2004). In response to DNA damage, PP2A dissociates from ATM, allowing autophosphorylation on S1981 (and several other residues) and conversion to an active monomer, which is facilitated by physical contact between ATM and the MRN complex at the site of the DSB.

In the context of ATM activation at the sites of DSBs, BRCA1-associated protein required for ATM activation-1 (BAAT1) serves to prevent the premature dissipation of ATM activity by binding to the activated ATM protein and preventing the premature dephosphorylation of ATM at serine-1981 by PP2A (Aglipay et al., 2006). Further activation of ATM is mediated by the chromatin-binding acetyltransferase TIP60 (Sun et al., 2010). TIP60 targets ATM by binding to trimethylated histones near the DSB and acetylating ATM within its PIKK regulatory domain (PRD), which lies adjacent to its kinase domain.

The scheme described above and illustrated in Figure 2A constitutes the classical activation mechanism for ATM in the setting of DNA damage. Recent studies indicate a second mechanism for ATM activation due to oxidative stress (Guo et al., 2010; Ditch and Paull, 2012). Here, ATM, which also mediates a cytoprotective response to oxidative stress, is activated by a direct mechanism through oxidation of the ATM protein, which does not require the MRN complex (Figure 2B). The result is an active ATM dimer held together by a disulfide linkage that contains two phosphorylated serine-1981 residues.

## ATM downstream signaling: recruitment of BRCA1 to DSBs

A large number of different substrates for ATM have been identified (Kastan and Lim, 2000), but herein we will focus on those most closely involved in the recruitment of BRCA1 to the sites of DSBs. In the setting of DNA damage, ATM very rapidly phos-phorylates a nearby variant histone (H2AX) on serine-139 (the phosphorylated form of H2AX is known as γ-H2AX), although it is clear that other kinases (e.g., ATM and Rad5-related, ATR) in different contexts can also phosphorylate H2AX (Burma et al., 2001; Wang et al., 2005). Phosphorylated H2AX is recognized by mediator of DNA damage checkpoint protein 1 (MDC1), allowing the recruitment of MDC1 to the sites of DSBs (Stewart et al., 2003; Lee et al., 2005). These events occur very rapidly (within seconds) following the formation of a DSB. MDC1, like H2AX is also a substrate for the ATM kinase. MDC1 serves as a scaffold for the accumulation of other DDR proteins at DNA damage sites and also functions to amplify the DDR (Lou et al., 2006). The proposed mechanism is that MDC1 bound to γ-H2AX can then recruit a second ATM, through the interaction of ATM with the FHA domain of MDC1. This allows phosphorylation of a second H2AX molecule and subsequent recruitment of another MDC1 molecule and so on (Lou et al., 2006; Yan and Jetten, 2008). Besides ATM, MDC1, and γ-H2AX, the MRN complex is also involved in this amplification process, through a mechanism in which MDC1 is phosphorylated by casein kinase 2 (CK2) and NBS1 binds to MDC1 through its phosphorylated CK2 site (Spycher et al., 2008). These mechanisms allow extension of the DNA damage signal up to 1 Mb upstream and downstream of the original break site and explains why ionizing radiation-induced DNA repair foci (IRIF) can be easily detected by immunofluorescence microscopy (Costes et al., 2010).

**Figure 3:**
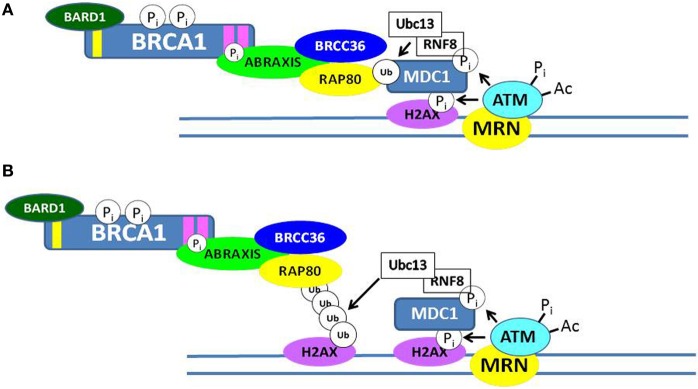
**Recruitment of BRCA1 to sites of double-strand DNA breaks**. Two possible mechanisms by which the BRCA1 complex A can be recruited to ionizing radiation-induced foci (IRIF) are illustrated in **(A,B)**. Both involve post-translational modifications of DDR proteins, including phosphorylation and ubiquitination. In **(A)**, the RNF8/Ubc13 complex ubiquitinates MDC1, and the ubiquitin-interacting motif (UIM) of RAP80 interacts with ubiquitinated MDC1. In **(B)**, RNF8/Ubc13 polyubiquitinates a nearby histone H2AX and the UIM of RAP80 interacts with the ubiquitinated H2AX protein. In each case, phosphorylated Abraxis interacts with the BRCT domain of BRCA1 and the RAP80 protein, thus recruiting BRCA1 to the site of the DSB.

Although BRCA1 is phosphorylated relatively rapidly in response to DSBs (see below), its recruitment in large quantities to IRIF is usually delayed (>1 h). Recent progress has elucidated several mechanisms by which BRCA1 becomes localized to IRIF (Kolas et al., 2007; Sobhian et al., 2007; Yan and Jetten, 2008; Strauss et al., 2011; Campbell et al., 2012; Mattiroli et al., 2012; Zhang et al., 2012). Two mechanisms are illustrated in Figure 3. In one scheme, the E3 ubiquitin ligase RNF8 binds to MDC1 in a phosphorylation-dependent interaction and along with an associated E2 ubiquitin-conjugating enzyme (Ubc13) ubiquitinates MDC1 on lysine-1977 of MDC1 (Figure 3A). Then RAP80, through its ubiquitin-interacting motif (UIM) binds to ubiquitinated MDC1. RAP80, a component of BRCA1 complex A (Figure 1), recruits BRCA1 to the IRIF through the adaptor protein Abraxis, which interacts directly with BRCA1. This interaction is mediated through binding of the BRCT domain with the pSPXF [phosphoserine-proline–(X = any amino acid)–phenylalanine]. Other components of BRCA1 complex A include BARD1, BRCC36, BRCC45, and NDA1 (MERIT40) (Fong et al., 2009; Wang, 2012). In the second scheme, RNF8 and a second ubiquitin ligase (RNF168) mediate a specific polyubiquitination of a nearby H2AX or H2A molecule through Ubc13, and the ubiq-uitin chain is recognized by the UIM of RAP80, leading to the recruitment of the BRCA1 complex A (Figure 3B). The possible functions of the BRCA1 complexes A, B, and C are considered below. We note here that the recruitment of these complexes to DNA damage sites is mutually exclusive, suggesting that the complexes function during different phases of the cell cycle and/or at different times during the DDR.

### BRCA1 functions within the DDR

In the context of the DDR, early clues to *BRCA1* gene function came from studies of *Brca1*-deficient fibroblasts and tumors, which exhibited evidence of extensive genomic instability, including a pattern of aneuploidy, centrosomal amplification, and chromosomal aberrations (Tirkkonen et al., 1997; Xu et al., 1999; Weaver et al., 2002). Consistent with these findings, Scully et al. (1997a,b) had reported that: (1) BRCA1 colocalizes with Rad51, a DNA recombinase, in nuclear foci during S-phase; and (2) following DNA damage, BRCA1 became phosphorylated and translocated to PCNA-positive DNA structures containing Rad51, and BARD1. Taken together, these findings suggested a role for BRCA1 as a caretaker gene involved in the monitoring and maintenance of genomic integrity. Other studies indicated that BRCA1-deficient cells showed increased sensitivity to IR (Shen et al., 1998; Scully et al., 1999; Ruffner et al., 2001). Since cell death following IR is mainly due to incomplete repair of DNA DSBs, these findings suggest a role for BRCA1 in the DDR pathways that are activated in response to DSBs.

### Role of BRCA1 in DNA damage-activated cell cycle checkpoints

Further research suggested specific roles for BRCA1 in response to DNA damage induced by IR. Thus, BRCA1 was found to be required for several DNA damage-responsive cell cycle checkpoints. These checkpoints are activated in response to DNA damage (e.g., DSBs) and function to block cell cycle progression in order to allow the repair of DNA lesions, so the damage is not propagated and passed on to daughter cells. One such BRCA1-regulated cell cycle checkpoint is the G2/M checkpoint (Xu et al., 2001; Yarden et al., 2002). Here, BRCA1 was found to be essential for the activation of checkpoint kinase 1 (CHK1), a key effector of G2/M arrest (Yarden et al., 2002). Both ATM (A-T mutated) and BRCA1 were found to be required for the IR-induced S-phase as well G2 checkpoints; and a specific phosphorylation of BRCA1 by ATM at serine-1423 was required for activation of the G2 the checkpoint (Xu et al., 2001). BRCA1, as well as the ATR protein, were also found to participate in another G2 cell cycle checkpoint known as the decatenation checkpoint (Deming et al., 2001). This checkpoint monitors the status of chromatid unwinding and delays cell entry into mitosis until the chromatids are sufficiently unwound (decatenated), in order to prevent chromosomal stress that might lead to aneuploidy or polyploidy.

The DNA damage-induced S-phase checkpoint results in inhibition of replication initiation in response to DNA damage. A defect in this checkpoint results in continued DNA synthesis, also called radioresistant DNA synthesis following IR. This checkpoint was found to require an ATM-dependent phosphorylation of BRCA1 on serine-1387 as well as a functional NBS1 (Xu et al., 2002). In addition to DNA damage-responsive checkpoints, several studies indicate that BRCA1 also regulates the mitotic spindle checkpoint by regulating gene expression associated with orderly progression through mitosis (Wang et al., 2004; Bae et al., 2005). Here, BRCA1 deficiency caused a defect in the spindle checkpoint (which ensures orderly separation of chromatids) as well as a defect in cytokinesis that resulted in accumulation of multinucleated cells, Several recent studies suggest that a CHK2-mediated phosphorylation of BRCA1 (see Figure 1) is required for orderly assembly of the mitotic spindle and proper segregation of chromosomes (Stolz et al., 2010a,b).

Finally, a role for BRCA1 in the IR-induced G1/S checkpoint, which blocks entry of cells containing chromosomal breaks into S-phase, has been demonstrated. Here, in response to IR, ATM phos-phorylates BRCA1 on serine-1423 and serine-1524, which allows the efficient ATM-mediated phosphorylation of p53 on serine-15, activation of p53 transcriptional activity, and subsequent expression of the cell cycle inhibitor p21 (Fabbro et al., 2004). In this study, the BRCA1/BARD1 complex was required for the ATM phosphorylation of p53 and subsequent G1/S cell cycle arrest.

### Role of BRCA1 in homology-directed DNA repair

Double-strand breaks can be repaired by two major pathways: (1) homology-directed repair (HDR; also called homologous recombination); or (2) non-homologous end joining (NHEJ) (Symington and Gautier, 2011). Here, we will consider the role of BRCA1 in HDR, while its putative role in NHEJ will be discussed in the next section. HDR can only occur during S-phase and G2-phase, because the homologous strand of the corresponding sister chromatid is required as a template for repair-related DNA synthesis. This form of DSB repair is usually considered to be error-free, and thus a mechanism for maintenance of genomic integrity (but see below). Moynahan et al. (1999) first described a major role for BRCA1 in HDR based on the finding that a Brca1-deficient reporter mouse embryonic stem (ES) cell line failed to accurately repair a chromosomal DSB created by the I-Sce 1 endonuclease. In related studies, the same group demonstrated that BRCA2 was also required for HDR and that the defect in HDR in Brca1-deficient cells could be corrected by either expression of a wild-type BRCA1 transgene or correction of one mutated Brca1 allele through gene targeting (Moynahan et al., 2001). While these studies definitively establish a role for BRCA1 in HDR, they do not address its biochemical function in HDR. One clue to this function is the demonstration of a requirement for an ATPase-competent RAD51 protein for HDR, which was not surprising since RAD51 is the mammalian homolog of the bacterial DNA recombinase RecA (Stark et al., 2002). It has been suggested that HDR is the major tumor suppressor function for both BRCA1 and BRCA2, since a deficiency in HDR leads to increased levels of NHEJ and single-strand annealing (SSA), both of which are error-prone processes that lead to genomic instability.

While the process of homologous recombination has been extensively investigated over several decades, the role of BRCA1 in this process has not been fully worked out. A review of this process and its potential role in tumor suppression can be found elsewhere (Moynahan and Jasin, 2010). The first step in HDR involves the 5′–3′ end resection of DNA starting at the site of the DSB. This resection creates a segment of single-stranded DNA (ssDNA) that can then invade the sister chromatid and pair with the complimentary DNA strand, allowing the initiation of repair. These resected ends can then be utilized by RAD51, which catalyzes the crossover reaction. In this regard, it is thought that BRCA1 in complex with the CtBP-interacting protein (CtIP; designated “complex C”) (Figure 1) facilitates the end resection by allowing the recruitment of replication protein A (RPA), a ssDNA-biding protein (Sartori et al., 2007; Buis et al., 2012; Escribano-Díaz et al., 2013). The phosphorylation of CtIP that is required for its recognition of and binding to the BRCT domains of BRCA1 is mediated by cyclin-dependent kinase 2 (CDK2) and is facilitated by MRE11, a component of the MRN protein complex (Buis et al., 2012); and both the MRN complex and CtIP were found to contribute to DNA end resection at the sites of DSBs (Sartori et al., 2007). Complex C also participates in the G2/M cell cycle checkpoint (Yu et al., 2006).

After end resection and recruitment of RPA to the newly created ssDNA, the recombinase RAD51 is recruited to the resected ends. The recruitment of RAD51 is dependent upon other proteins, including RAD54 and BRCA2 (which directly binds multiple copies of RAD51 copies and regulates their activity) (Gudmundsdottir and Ashworth, 2006). Partner and localizer of BRCA2 (PALB2) is required for the localization of BRCA2 at DNA damage sites and its participation in HDR and, in turn, PALB2 binds directly to BRCA1, suggesting that it functions as an adapter between BRCA1 and BRCA2 during the process of HDR (Sy et al., 2009; Zhang et al., 2009; Buisson and Masson, 2012) (see Figure 1). The final steps in DSB repair by HDR involves the formation two Holliday junctions, which are then resolved without crossover, restoring the DNA to its original condition without sequence abnormalities (Moynahan and Jasin, 2010). It is noted here that while both BRCA1 and BRCA2 function in the HDR pathway, the role of BRCA1 in the DDR is broader than that of BRCA2, since BRCA1 also mediates cell cycle checkpoints.

### Functions of BRCA1 complexes A, B, and C

Although HDR is generally considered to be an error-free process, an aberrant error-prone form of homologous recombination called “hyper homologous recombination” (HHR) has been described (Harris and Khanna, 2011; Dever et al., 2012). HHR was observed in the presence of mutant forms of BRCA1 (e.g., M1775R) that disrupt the interaction of the BRCT domain with phosphopeptide sequences or when components of complex A (Abraxas, RAP80, or BRCC36) were knocked down (Figure 1). It has been suggested that BRCA1 complex A functions, in part, as a de-ubiquitinating complex to limit end resection during the early stages of HDR to prevent excessive accumulation of RAD51 and RPA on the invading DNA strand. Other studies indicate that complex A also participates in the G2/M cell cycle checkpoint and in localizing BRCA1 to IRIF (Kim et al., 2007a,b; Wang et al., 2007a).

The BRCA1 complex B consists of BRCA1 and BACH1 (BRCT helicase; also known as BRIT and FANCJ) and is formed by the interaction of the BRCA1 BRCT with phosphoserine-990 of BACH1, which is part of a pSPXF motif (Cantor et al., 2004; Peng et al., 2006; Kumaraswamy and Shiekhattar, 2007; Gong et al., 2010; Tomimatsu et al., 2012). The function of complex B is not as clear, but it has been suggested that complex B is required for orderly progression through S-phase, including the bypassing of stalled replication forks, and also serves a DNA repair function that is not well defined. Complex C (BRCA1-CtIP-MRN) is formed through the interaction of the BRCA1 BRCT domain with phosphoserine-327 of CtIP, which is also part of a pSPXF motif. This complex is thought to stimulate DNA end resection by MRE11 during DNA repair by HDR (reviewed in Wang, 2012). However, another nuclease, EXO1, may play a more important role in DNA end resection during DSB repair (Tomimatsu et al., 2012). Knockdown of components of complex A or C cause a defect in the G2/M cell cycle checkpoint, but the defect created by disruption of complex C is more mild than that created by knockdown of complex A. The molecular details of how these complexes function, their precise roles in the maintenance of genomic integrity, and the mechanisms by which they are assembled and disassembled remain to be determined. Additional information on the BRCA1 complexes and their significance can be found elsewhere (Wang, 2012).

### Role of BRCA1 in non-homologous end joining

Non-homologous end joining involves a very different set of proteins from HDR [e.g., Artemis, XRCC4, DNA polymerase lambda, DNA ligase IV (LIG4), and DNA-dependent protein kinase (DNA-PK)] and, unlike HDR, occurs predominantly during G1 and less so in S-phase or G2 (Lieber, 2010; Dever et al., 2012). HDR cannot occur during G1, because a homologous segment of DNA that can act as a template for repair synthesis is unavailable in G1. The significance of this process is that it can be an error-prone process because of modification of the broken DNA ends, which can result in short or longer deletions. With regard to the DDR, cells defective for NHEJ show hypersensitivity to IR, suggesting that NHEJ is a major pathway for repair of DSBs generated by IR. The literature on the putative role of BRCA1 in NHEJ is unsettled, because several studies suggest a requirement for BRCA1 in NHEJ (Baldeyron et al., 2002; Zhong et al., 2002a,b; Bau et al., 2004), while others find no defect in NHEJ in BRCA1-deficient cells (Moynahan et al., 1999; Wang et al., 2001; Mérel et al., 2002). While the role of BRCA1 in NHEJ remains controversial, a suggested explanation is that there are several forms of NHEJ, including one that is error-prone and another that is relatively precise; and BRCA1 only promotes the precise end joining (Durant and Nickoloff, 2005; Gudmundsdottir and Ashworth, 2006). The presumed mechanism is that BRCA1, when bound to DNA, inhibits the nuclease activity of MRE11 or the MRN complex, thus limiting DNA end resection (Durant and Nickoloff, 2005).

**Figure 4:**
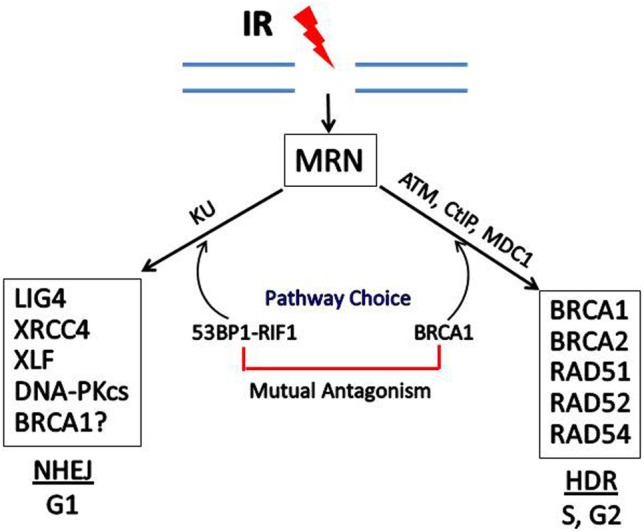
**Double-strand break repair by homology-directed repair (HDR) or non-homologous end joining (NHEJ)**. In response to IR, the MRN complex recognizes and binds to the broken ends of DNA at DSBs. DSB repair can occur by NHEJ or HDR, depending upon the phase of the cell cycle and the relative levels of BRCA1 vs. 53BP1 which has been phosphorylated by ATM and is complexed with RIF1. Some of the proteins involved in NHEJ and HDR are shown in the boxes. In addition to HDR, BRCA1 may participate in one subtype of NHEJ, but the role of BRCA1 in NHEJ is still not certain.

The participation of BRCA1 in the choice and execution of DSB DNA repair pathways is illustrated in Figure 4. Recent studies have identified a cell cycle-dependent mechanism that underlies the DNA DSB repair pathway choice. p53 Binding protein 1 (53BP1), when phosphorylated by ATM, binds to RAP1-interactibg factor 1 (RIF1) and recruits RIF1 to DSB sites, where it inhibits 5′ end resection required for HDR, thus promoting the NHEJ pathway. In contrast, BRCA1 promotes 5′ end resection and thus HDR (Escribano-Díaz et al., 2013; Feng et al., 2013). BRCA1 expression is normally low in G1 and increases significantly during S-phase and G2. The accumulation of BRCA1 during S and G2 counteracts the ability of 53BP1-RIF1 to stimulate NHEJ. In contrast, when BRCA1 levels are low during G1, 53BP1-RIF1 accumulate at DSBs unopposed by BRCA1, resulting in NHEJ being the predominant pathway for DSB reverse during G1.

### BRCA1/BARD1 and its ubiquitin ligase function

Breast and ovarian cancer susceptibility gene 1-associated ring domain 1 was first identified as a RING domain protein that interacts and colocalizes with BRCA1 through a RING: RING interaction involving the N-termini of each protein (Wu et al., 1996; Jin et al., 1997) (Figure 1). Several cancer-associated point mutations of the RING domain of BRCA1 (e.g., Cys61Gly and Cys64Gly) disrupted the BRCA1: BARD1 interaction, suggesting that this interaction contributes to BRCA1-dependent tumor suppression. An early functional study suggested that BARD1 in association with CstF-50 plays a role in regulation of RNA processing during transcription by inhibiting polyadenylation (Kleiman and Manley, 1999); and a subsequent study suggested that this function may be linked to DNA repair (Kleiman and Manley, 2001). A significant advance in understanding the physiologic importance of the BRCA1: BARD1 interaction was the finding that the BRCA1: BARD1 heterodimer functions as an E3 ubiquitin ligase and that this ubiquitin ligase activity was abolished by cancer-associated mutations within the BRCA1 RING domain (Brzovic et al., 2001; Hashizume et al., 2001). These findings led to the hypothesis that many of the functions of BRCA1, including its tumor suppressor activity, were due to or required the ubiquitin ligase activity of the BRCA1: BARD1 complex (Baer and Ludwig, 2002). Further study suggested that the BARD1 interaction with BRCA1 is required for HDR of chromosomal breaks (Westermark et al., 2003). This finding coupled to the observation of cancer-associated BRCA1-mutations that disrupt the association of BRCA1 with the E2 ubiquitin-conjugating enzyme UbcH5 (Morris et al., 2006) and the finding that cancer-associated RING domain mutations of BRCA1 that disrupt the ubiquitin ligase function cause hypersensitivity to IR (Ruffner et al., 2001). These considerations led to a great interest in identifying the *in vivo* targets of the BRCA1: BARD1 ubiquitin ligase (Wu et al., 2008).

Then, in 2008, Ludwig and his colleagues generated an isogenic set of murine ES cells that expressed either wild-type *Brca1* or a mutant *Brca1* (I26A) that lacks E3 ubiquitin ligase activity but retains the ability to bind to Bard1 (Reid et al., 2008). Surprisingly, not only were the *Brca1* I26A mutant ES cells viable, but they also exhibited normal sensitivity to the DNA cross-linking agent mit-omycin C, formed RAD51 foci in response to IR, and exhibited wild-type rates of HDR (Reid et al., 2008). These findings challenged the prevailing view that the BRCA1: BARD1 E3 ubiquitin ligase activity, the only known enzymatic function of BRCA1, was required for most major functions of BRCA1 thought to be critical for tumor suppression. In a subsequent study, the same investigators demonstrated that transgenic mice homozygous for mutant *Brca1* I26A targeted to specific tissues (e.g., pancreas or mammary gland) suppressed tumor formation to the same degree as wild-type *Brca1;* whereas a *Brca1*-mutation of the BRCT domain that abrogated phosphoprotein-binding (S1598F) conferred a high rate of tumor formation in the same genetic models (Shakya et al., 2011). The investigators concluded that the ubiquitin ligase function of BRCA1 was dispensable for tumor suppression, while the recognition of phosphoproteins by the BRCT domains of BRCA1 was essential for suppression of tumor formation.

These findings have still not finally settled the question of the role of the BRCA1 ubiquitin ligase function in tumor suppression. For example, it was recently reported that BRCA1 normally plays a major role in repressing satellite DNA (Zhu et al., 2011). Satellite DNA consists of long stretches of non-coding DNA characterized by tandemly repeated sequences; and it is a major component of heterochromatin. In this study, BRCA1 deficiency led to the loss of transcriptional repression of satellite DNA in mouse mammary tumors, human breast cancers, and cultured cells and to loss of ubiquitinated histone H2A within the satellite repeats. Furthermore, BRCA1 was shown to bind to satellite DNA and ubiquitinate H2A. The BRCA1-deficient phenotype was reversed by ectopic expression of an H2A-ubiquitin protein. Conversely, this phenotype (including genomic instability associated with defects in HDR and cell cycle checkpoints) was reproduced by the ectopic expression of satellite RNA. The authors' conclusion that most BRCA1 tumor suppressor functions are due to its role in the maintenance of heterochromatin structure (Zhu et al., 2011) is inconsistent with the idea that the BRCA1 ubiquitin ligase function is dispensable for tumor suppression (Shakya et al., 2011).

Indirect evidence regarding BRCA1 tumor suppressor function comes from a study by Gayther et al. (1995) who described a genotype-phenotype correlation with location of the mutation within the *BRCA1* gene, in *BRCA1* breast and/or ovarian cancer families. Examination of the ratio of breast/ovarian cancer revealed that mutations that mapped to the N-terminus of the BRCA1 protein (including missense mutations and protein-truncating mutations that deleted the BRCT domains) exhibited a higher ratio of breast/ovarian cancers than did mutations mapping to the C-terminal portion of the *BRCA1* gene. These findings suggest that BRCA1 proteins missing the BRCT domains (and thus defective for HDR) can still suppress development of ovarian cancer. Other interpretations of these data are possible, but they do suggest differences in the mechanisms for development of breast vs. ovarian cancer in *BRCA1*-mutation carriers.

Moreover, the idea that *BRCA1* deficiency causes cancer solely due to genomic instability associated with the loss of HDR and cell cycle checkpoints does not account for the limited spectrum of tumor types observed in BRCA1-mutation carriers. Thus, a study of nearly 700 *BRCA1* families indicates that breast and ovarian cancers are by far the most common, while there is a higher than expected risk in several additional hormonally related cancers, including cervical cancer, uterine cancer, and prostate cancers in younger men (Thompson et al., 2002). These considerations suggest that BRCA1 exerts another function(s), perhaps endocrine-related, that collaborates with its role in maintenance of genomic integrity to explain why BRCA1-mutations lead to a specific set of tumor types. Thus, BRCA1 was found to inhibit estrogen receptor activity, both in cultured cells and mouse models; and accumulating evidence suggest that BRCA1-related tumorigene-sis is a hormonally responsive process, both in mice and humans (reviewed in Rosen et al., 2005).

Finally, the finding that the ubiquitin ligase function of the BRCA1: BARD1 is not required for HDR or tumor suppression in the mouse raises the additional question of why missense mutations of BRCA1 that disrupt the BRCA1: BARD1 interaction (e.g., Cys61Gly) lead to cancer in humans and in mice (Drost et al., 2011). These considerations would suggest that the BRCA1: BARD1 interaction may have another ubiquitin ligase-independent function that is essential for tumor suppression. Interestingly, BARD1 is itself a tumor suppressor, mutations of which have been linked to breast, ovarian, and endometrial cancers (Ghimenti et al., 2002; Sauer and Andrulis, 2005). However, curiously, some of the cancer-associated mutants of BARD1 do not alter the function of BARD1 in HDR (Laufer et al., 2007).

### Role of p53 in *BRCA1*-dependent tumorigenesis

As noted above, BRCA1 can interact directly with p53 and stimulate its transcriptional activity. Interestingly, in studies of human BRCA1-related cancers, the incidence of p53 mutations (over 80%) is considerably higher than in sporadic breast cancers (25%) (Phillips et al., 1999; Holstege et al., 2009). Studies of *Brca1* knockout mice revealed early embryonic lethality, usually by day 7.5 (Hakem et al., 1996). The Brca1–/– phenotype was characterized by widespread defects in cell proliferation due, in part, to p53 activation. This phenotype was partially reversed by a p53 or p21 deficiency, resulting in embryonic death at later times (Hakem et al., 1997). It was suggested that p53 was activated due to chromosomal abnormalities created by *Brca1* deficiency, causing p53 activation and p21 expression, resulting in cell cycle arrest or senescence. By the same reasoning, it appears that a p53 mutation is required for BRCA1-related breast cancer development because otherwise, chromosoml aberrations due to *BRCA1* deficiency would activate p53, leading to cell cycle arrest, apoptosis, and/or senescence of the tumor cells.

In a recent study, it was found that p53 mediates the nuclear export of wild-type BRCA1 via a BRCA1: p53 protein interaction and possibly, in part, by disrupting the BRCA1: BARD1 interaction (Jiang et al., 2011). It was suggested that this mechanism could increase cellular sensitivity to DNA damaging agents such as IR and that loss of p53 function could impair the nuclear export of BRCA1 in sporadic breast cancers with functional BRCA1, resulting in greater resistance to DNA damaging agents. Thus, the functional interaction of BRCA1 and p53 is quite complex and may influence the molecular pathogenesis of breast cancer, the DDR of tumor cells, and their sensitivity to DNA damaging agents including chemotherapy drugs and IR.

### BRCA1 and telomeres

Telomeres, the ends of chromosomes that contain varying lengths of hexameric DNA repeats (TTAGGG in mammalian cells) are of interest in the context of DNA repair for several reasons: (1) if chromosome ends were recognized as DSBs, it would lead to genomic instability due to end joining and translocations; (2) conversely, telomerase is recruited to internal DSBs, where it could potentially generate a telomere, with disastrous consequences; (3) DNA damage can cause telomere shortening; and (4) telomere shortening can lead to chromosomal instability and cancer development (Günes and Rudolph, 2013; Ribeyre and Shore, 2013). Moreover, tumor cells that do not express telomerase, the enzyme complex that adds TTAGGG to telomeres, utilize alternative lengthening of telomeres (ALT), a method of telomere maintenance that involves DNA recombination and utilizes some of the same DNA repair proteins involved in repairing DSBs (Nandakumar and Cech, 2013). To start out, telomeres are protected from being recognized as DSBs, in part, by a complex of six intrinsic telomeric proteins known collectively as “shelterin” (TRF1, TRF2, TIN2, TPP1, POT1, and RAP1). Three of these proteins directly bind telomeric DNA (TRF1, TRF2, and POT1); and the other three proteins (TIN2, TPP1, and RAP1) do not directly contact DNA but serve to interconnect the three DNA-binding proteins. The shelterin proteins contribute to the formation of the telomere loop (t-loop) at the end of the chromosome and inhibit the activity of ATM and ATR (Raffaella Diotti and Loayza, 2011). In particular, TRF2 and POT1 have been implicated in the inhibition of ATM and ATR, respectively. Other proteins can bind transiently to shelterin to alter telomere function.

**Figure 5:**
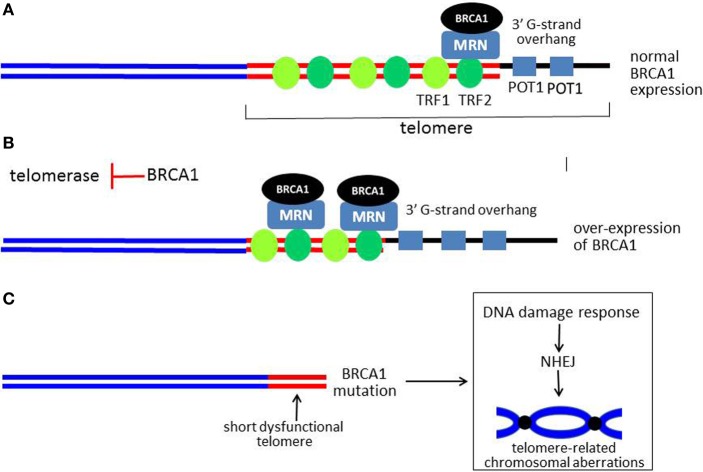
**Model for role of BRCA1 in telomere maintenance. (A)** Shows a linear representation of a normal functional telomere. For simplicity, not all of the telomere-associated proteins are shown. BRCA1 is recruited to the telomere by RAD50, a component of the MRN complex, which is also present at the telomere. When BRCA1 is over-expressed, more BRCA1 is present at the telomere. BRCA1 causes overall telomere shortening, but the 3′ G-strand overhang is lengthened, as illustrated in **(B)**. **(C)** Shows a critically short and dysfunctional telomere with little or no 3′ G-strand overhang in cells with no functional BRCA1. A DDR is activated with resultant chromosomal aberrations due to end-end fusions and translocations (a dicentric chromosome is illustrated). The G-strand overhang is represented by a thick black line. The thick red lines represent double-stranded telomeric DNA, while the sub-telomeric DNA is shown as blue lines.

One group of proteins that bind to shelterin is the MRN complex. In this context, the same MRN complex which initiates the repair of DSBs, when recruited to the telomere by TRF2 has been implicated in regulation of telomere length and the telomeric overhang (Lamarche et al., 2010) (Figure 5A). MRN as well as the nuclease Apollo have been implicated in generation of the telomeric overhang (a 3′ G-rich single-stranded telomeric extension), which functions in maintaining telomere stability. However, far less is known about how telomeric MRN functions than how MRN functions in the sensing and repair of DSBs. As in the case of the response to DSBs, ATM is required for MRN signaling in the context of a dysfunctional uncapped telomere (Lamarche et al., 2010). In this setting a signaling cascade similar to that induced by DSBs is activated and can result in cell cycle arrest, senescence, or apoptosis.

In addition to MRN, several studies implicate BRCA1 as a regulator of telomere length and stability. In the first study, over-expression of BRCA1 was found to inhibit telomerase enzymatic activity by transcriptionally repressing expression of the telom-erase catalytic subunit (telomerase reverse transcriptase, TERT) (Xiong et al., 2003). The mechanism of repression appears to be by inhibiting the ability of the c-Myc oncoprotein to stimulate TERT expression through the c-Myc E-box within the TERT proximal promoter. As a consequence of telomerase inhibition, over-expression of wild-type BRCA1 but not a cancer-associated mutant (Cys61Gly) caused telomere shortening in several tumor cell lines (Figure 5B). Interestingly, despite causing shortening of telomeres to very small sizes (well under 2.0 kb), BRCA1 did not cause inhibition of cellular proliferation, cell cycle arrest, senescence, or apoptosis (Xiong et al., 2003). Surprisingly, significant telomere shortening due to BRCA1 occurred very rapidly (within 2–3 cell doublings), far too fast to be attributable to inhibition of telomerase activity. These findings suggest that BRCA1 causes telomere erosion (degradation) but somehow protects against telomeric dysfunction.

In a second study, it was found that BRCA1 knockdown resulted in increased telomerase activity and significant telomere lengthening in tumor cells (Ballal et al., 2009). Based on telomeric chromatin immunoprecipitation (telomeric ChIP) assays, the presence of BRCA1 on telomeres was documented. BRCA1 was also found to interact and colocalize with shelterin proteins TRF1 and TRF2, in DNA-dependent manner. In further studies, it was found that BRCA1 was recruited to the telomere by RAD50, a component of the MRN complex. Finally, it was found that, like MRN, BRCA1 regulates the length of the 3′ G-rich overhang. Thus over-expression of BRCA1 caused lengthening and knockdown of BRCA1 or RAD50 caused a similar degree of shortening of the 3′ overhang (Figure 5B). These findings suggest that BRCA1 can regulate both telomere length and telomere stability and may mediate some of the effects of the MRN complex on the telomere (e.g., overhang length). These findings are consistent with the observation that cells with no functional BRCA1 exhibit evidence of telomere dysfunction and loss of the capping function, evidenced by very short telomeres and the appearance of chromosomal abnormalities of the type expected from telomere dysfunctions (e.g., dicentric chromosomes due to end-end fusion) (Al-Wahiby and Slijepcevic, 2005; McPherson et al., 2006; Wang et al., 2007b) (Figure 5C). In another study, knockdown of BRCA1 in a mammary epithelial cell line caused chromosomal aberrations consistent with telomere dysfunction (e.g., anaphase bridges). In addition to BRCA1 and MRN, defects of other DDR-associated proteins (Ku, DNA-PKcs, and RAD51D) have been linked to the loss of the telomeric capping function (Cabuy et al., 2008).

Finally, in understanding the relationship between telomeres and the DDR, it was mentioned above that telomerase is recruited to DSBs, where under some conditions it can synthesize telom-eres at the broken ends of DNA (Ribeyre and Shore, 2013). The discovery of telomeric DNA sequences within the interiors of chromosomes interstitial telomeric sequences (ITS) of rodents and primates has been interpreted to mean that at some time during evolution telomerase was utilized to repair DSBs (Slijepcevic, 2006). Based on mutational analysis, several studies have demonstrated the existence of active mechanisms in yeast to prevent the synthesis of telomeres by the enzyme telomerase at DNA ends of DSBs (Nergadze et al., 2007; Makovets and Blackburn, 2009; Zhang and Durocher, 2010). For example, Mec1 (the ortholog of ATR in yeast) both recognizes DNA ends and inhibits telomerase at DSBs, a mechanism for the preservation of genomic integrity. Two such mechanisms involve Mec1-dependent phosphorylation of Pif1 (a telomerase inhibitor) and Cdc13 (a telomere capping protein) (Makovets and Blackburn, 2009; Zhang and Durocher, 2010). Genetic analysis in *Drosophila* identified ATR-interacting protein (ATRIP) as a factor involved in preventing the formation of telomeres at the sites of DSBs (Beaucher et al., 2012). In mammalian cells, ATR: ATRIP complexes are recruited to ssDNA coated with RPA and are activated by a complex mechanism that is not fully understood (Liu et al., 2011). In this context, ATR: ATRIP complexes have been found to activate a CHK1-dependent checkpoint mechanism during S-phase in response to stalled replication forks (Nam and Cortez, 2011).

### Other DNA damage-related functions of BRCA1

While the best studied DNA damage-related function of BRCA1 is its role in the repair of DSBs, roles for BRCA1 in other DNA repair processes have been reported. Thus, BRCA1 has been reporter to up-regulate the activity of the base excision repair (BER) pathway through a transcriptional mechanism that involves stimulation of the expression of several key BER enzymes (OGG1. NTH1, and REF1/APE1) (Saha et al., 2010a,b). BER is the major pathway for the repair of oxidized DNA and is normally an error-free process. Failure to repair different types of oxidized DNA lesions can result in cytotoxicity or mutagenesis, which can ultimately lead to cancer. The mechanism for up-regulation of BER enzyme expression was identified as stimulation of the activity of the OCT1. Previously, it was shown that BRCA1, like ATM, mediates a cytoprotective antioxidant response, characterized by stimulation of the activity of the antioxidant response transcription factor NFE2L2 (NRF2) (Bae et al., 2004). Further studies have revealed that BRCA1, in collaboration with REF1, down-regulates intracellular levels of reactive oxygen species (ROS), oxidized DNA, and nitrated proteins (Saha et al., 2009). However, the contribution of these functions of BRCA1 to tumor suppression is unknown.

The Fanconi anemia network consists of a group of proteins involved in the repair of DNA interstrand cross-links (ICLs), mutations of which lead to Fanconi anemic, a genetic disorder characterized by short stature, chromosomal instability, bone marrow failure, and increased sensitivity to agents that cause cross-linking of DNA. The accurate repair of ICLs involves, in part, HDR as well as the nucleotide excision repair (NER) pathway. Several studies suggest that BRCA1 participates in the repair of ICLs. These studies suggest two distinct roles for BRCA1 in ICL repair, one involving its function in HDR and the other independent of HDR (Zhou et al., 2005; Cheng et al., 2006; Bunting et al., 2012). However, the precise molecular functions of BRCA1 in the repair of ICLs are unclear. Conversely, as described above, several components within the Fanconi anemia network, interact with BRCA1 (directly or indirectly) and participate in BRCA1-dependent DNA repair of DSBs, including FANCJ (=BACH1), FANCN (=PALB2), and FANCD1 (BRCA2).

In addition to ATM, BRCA1 is also phosphorylated at several sites by ATR in response to ultraviolet (UV) radiation (Tibbetts et al., 2000; Gatei et al., 2001). These findings suggest a role for BRCA1 in the repair of UV damage, which is mediated, in part, by the NER pathway. In a recent study, it was reported that BRCA1 participates in the response to UV damage in a manner that is independent of the NER pathway (Pathania et al., 2011). Here it was found that following UV damage, BRCA1 is recruited through its BRCT domains to stalled replication forks, where it participates in several processes including excision of photoproducts and recruitment of components of the replication factor C (RFC) complex, with subsequent checkpoint activation and post-replicative DNA repair. Unlike BRCA1 recruitment to IRIF (which is delayed for more than 1 h), BRCA1 is recruited relatively rapidly (15 min) to sites of UV damage, primarily in S-phase cells (Pathania et al., 2011; Zhang et al., 2013). In one study, it was reported that BRG1, a component of the SWI/SNF chromatin remodeling complex, is required for BRCA1 recruitment to UV damage sites and that BRG1 modulates BRCA1 function in repair of UV damage by regulating the activation of ATR and ATM. Several other studies suggest roles for BRCA1 in the repair of UV damage (Navaraj et al., 2005; Marteijn et al., 2009) and it has been proposed that BRCA1 transcriptionally up-regulates genes involved in NER (Hartman and Ford, 2002).

### BRCA1 and PARP

Poly(ADP-ribose) polymerase (PARP) is a nuclear enzyme in the BER pathway that participates in the repair of single-strand breaks (SSBs) of DNA. Using a “synthetic lethal” screen, it was found that inhibition of PARP activity causes chromosomal instability and apoptosis in *BRCA1* or BRCA2-mutant cells but not in *BRCA1/BRCA2*-competent cells (Farmer et al., 2005). It was hypothesized that inhibition of PARP causes the accumulation of unrepaired SSBs that are then converted DSBs that would normally be repaired by HDR. This observation has led to clinical trials of small molecule PARP inhibitors (which had been originally developed as chemosensitizers) as a treatment for tumors arising in *BRCA1* and *BRCA2* mutation carriers (Lord and Ashworth, 2008). In a phase I trial of the PARP inhibitor olaparib (formerly AZD2281), significant responses were observed only in *BRCA1*-mutant and *BRCA2*-mutant cancers, with no responses in tumors wild-type for *BRCA* (Fong et al., 2009). In another phase I trial, 40% of patients with ovarian cancers due to germ-line *BRCA1/2* mutations achieved complete or partial responses with olaparib, with the response rates higher in cis-platinum sensitive tumors than in cis-platinum resistant tumors (Fong et al., 2010). Currently, there are eight PARP inhibitors under clinical investigation (with more under development) either as monotherapy, in combination with chemotherapy and radiotherapy, or in combination with other specifically targeted agents, for various types of malignancies (Papeo et al., 2013).

It should be noted that although PARP inhibitors still hold great promise in cancer therapy, *de novo* resistance or the development of resistance after an initial response has become problematic (reviewed in Montoni et al., 2013). For example, in a recent phase II trial, none of 26 patients with advanced triple negative breast cancer (tumors that lack estrogen and progesterone receptor expression and do not exhibit amplification of the HER2/Neu oncogene) had objective responses to the PARP inhibitor olaparib (Gelmon et al., 2011). In contrast a response rate of 41% was observed in ovarian cancer patients who carry *BRCA1* or *BRCA2* mutations (Gelmon et al., 2011). One very interesting study of tumor tissue derived from patients with *BRCA2* mutations that had initially responded to olaparib but subsequently developed resistance revealed secondary mutations in the resistant tumors that restored BRCA2 function (Barber et al., 2013). In a mouse model with a knock-in cancer-associated *Brca1* mutation-Cys61Gly), the tumors rapidly developed resistance to both olaparib and cis-platinum but retained the *Brca1* mutation.

### Conclusions and perspectives

Breast and ovarian cancer susceptibility gene 1 is a tumor suppressor gene, inherited mutations of which confer a significantly increased risk breast and ovarian cancers. BRCA1 functions in the error-free repair of DSBs of DNA by HDR (also known as homologous recombination). This function appears to be critical for its tumor suppressor activity. BRCA1 may also function in a subtype of non-homologous end joining that is more accurate than classical error-prone NHEJ, although the role of BRCA1 in NHEJ is not settled. BRCA1 participates in a number of DNA damage-activated cell cycle checkpoints (e.g., intra-S and G2/M checkpoints) and in the response to stalled replication forks (e.g., those caused by DNA cross-linking agents). BRCA1 in complex with BARD1 exerts an E3 ubiquitin ligase activity that was once thought to be essential for tumor suppression, but this view was contradicted by a recent study of a transgenic mouse model homozygous for an engineered mutant *Brca1* gene that is defective for ubiquitin ligase activity but retains the ability to mediate HDR, since these mice did not develop cancer.

Since BRCA1 expression is widespread, the function of BRCA1 in mediating HDR and other DNA repair processes does not by itself explain the predilection of *BRCA1*-mutation carriers to develop such a limited range of tumor types, mostly breast and ovarian cancers. It is well-established that breast cancer is an estrogen-driven tumor type. Thus, the ability of BRCA1 to inhibit estrogen receptor activity (described above) could contribute to breast cancer suppression. Here, the idea is that during tumor development, mammary epithelial cells that exhibit genomic instability are stimulated to proliferate excessively because they lack a major mechanism that limits estrogen-stimulated proliferation. This hypothesis is consistent with findings suggesting that *BRCA1*-related tumorigenesis is hormonally responsive, at least in the early stages. It is expected that BRCA1 also mediates an ovary-specific function that could explain why the ovary is a preferred site for cancer development in women who carry *BRCA1* mutations.

It was also proposed that BRCA1 functions, including tumor suppression, can be explained by the ability of BRCA1 to ubiq-uitinate the histone H2A within satellite DNA, thus maintaining heterochromatin in a transcriptionally silenced state. Moreover, a clinical-epidemiologic study suggests that mutations mapping to the C-terminal region of BRCA1, which would be predicted to disrupt BRCA1 function in HDR, do not abrogate the ability of BRCA1 to suppress ovarian cancer. Thus the mechanisms by which BRCA1 suppresses breast and ovarian cancer development may differ. It is worthwhile to note that while mouse models of *Brca1*-dependent mammary tumorigenesis yield tumors with many of the characteristics of the human cancers, these models do not fully mimic the human situation. In addition to the obvious differences between mice and humans, *BRCA1*-related tumorige-nesis in mouse and humans differ in other characteristics. Thus, in mice, a homozygous *Brca1*-mutation is targeted to the mammary gland or other organs, often along with a heterozygous or homozygous deletion of p53. On the other hand, humans inherit one mutant *BRCA1* allele, with the other allele being wild-type. And since the mutation is in the germ-line, all somatic cells are heterozygous for *BRCA1*. Thus, the pathway for *BRCA1*-related tumorigenesis may not be the same in humans and mice.

We have reviewed evidence that BRCA1 serves other DNA damage-related functions, including the regulation of telomere length and stability. Consistent with other DDR proteins that have complex roles in telomere biology (e.g., MRN, ATM, DNA-PK, and others), BRCA1 exerts multiple telomere-related functions. Thus, BRCA1 inhibits telomerase activity and causes telomere shortening, while at the same time preserving telomere stability by increasing the length of the G-strand overhang. As a result, extremely short telomeres in BRCA1 over-expressing tumor cells did not trigger senescence, apoptosis, or cell cycle arrest. Although the telomerase activity is reduced in BRCA1 over-expressing cells, it apparently remains sufficient to maintain the length of the shortened telomeres and synthesize the 3′ G-strand overhang.

The mechanism by which BRCA1 causes telomere shortening remains to be determined, since the rate of shortening was too rapid to be due to the loss of telomerase activity alone. The complete absence of functional BRCA1 creates telomere dysfunction, evidenced by the appearance of chromosomal aberrations of the type due to critical telomere shortening. However, assuming that BRCA1 functions similarly in non-tumor cell types, the ability of BRCA1 to inhibit telomerase activity and cause telomere shortening are consistent with a tumor suppressor function. To what extent these activities actually contribute to the tumor suppressor activity of BRCA1 is unclear at present.

Finally, while much progress has been made in understanding how BRCA1 is recruited to IRIF and its function during the DDR, its precise molecular functions in HDR and other DNA repair activities (e.g., NEHJ and ICL cross-link repair) remain to be determined. Furthermore, in understanding the role of HDR in *BRCA1*-mediated tumor suppression, it should be realized that mutations that disrupt this function are likely to disrupt many other functions of BRCA1; and at present, it is unclear which of these other functions contribute to tumor suppression and to what extent.

### Conflict of interest statement

The authors declare that the research was conducted in the absence of any commercial or financial relationships that could be construed as a potential conflict of interest.
